# Indoor Air Quality at an Urban Primary School in Madrid (Spain): Influence of Surrounding Environment and Occupancy

**DOI:** 10.3390/ijerph21101263

**Published:** 2024-09-24

**Authors:** Elisabeth Alonso-Blanco, Francisco Javier Gómez-Moreno, Elías Díaz-Ramiro, Marcos Barreiro, Javier Fernández, Ibai Figuero, Alejandro Rubio-Juan, Jesús Miguel Santamaría, Begoña Artíñano

**Affiliations:** 1 Center for Energy, Environmental and Technological Research (CIEMAT), Avenida Complutense 40, 28040 Madrid, Spain; fj.gomez@ciemat.es (F.J.G.-M.); elias.diaz@ciemat.es (E.D.-R.); javier.fernandez3@ciemat.es (J.F.); b.artinano@ciemat.es (B.A.); 2 Regional Center for Animal Selection and Reproduction (CERSYRA), Ministry of Agriculture and Environment of Castilla-La Mancha, Avenida del Vino, 2, 13300 Valdepeñas, Spain; 3 Biodiversity and Environment Institute (BIOMA), Universidad de Navarra, Irunlarrea No. 1, 31008 Pamplona, Spain; chusmi@unav.es

**Keywords:** indoor air quality, indoor/outdoor ratio, primary school, black carbon, ultrafine particle number concentration, particulate matter

## Abstract

Monitoring indoor air quality (IAQ) in schools is critical because children spend most of their daytime inside. One of the main air pollutant sources in urban areas is road traffic, which greatly influences air quality. Thus, this study addresses, in depth, the linkages of meteorology, ambient air pollution, and indoor activities with IAQ in a traffic-influenced school situated south of Madrid. The measurement period was from 22 November to 21 December 2017. Simultaneous measurements of indoor and outdoor PM_1_, PM_2.5_, and PM_10_ mass concentrations, ultrafine particle number concentration (PNC) and equivalent black carbon (eBC) were analyzed under different meteorological conditions. PNC and eBC outdoor concentrations and their temporal trend were similar among the sampling points, with all sites being influenced in the same way by traffic emissions. Strong correlations were found between indoor and outdoor concentrations, indicating that indoor pollution levels were significantly affected by outdoor sources. Especially, PNC and eBC had the same indoor/outdoor (I/O) trend, but indoor concentrations were lower. The time delay in indoor vs. outdoor concentrations varied between 0.5 and 2 h, depending on wind speed. Significant differences were found between different meteorological conditions (ANOVA *p*-values < 2.14 × 10^−6^). Atmospheric stability periods led to an increase in indoor and outdoor pollutant levels. However, the highest I/O ratios were found during atmospheric instability, especially for eBC (an average of 1.2). This might be related to rapid changes in the outdoor air concentrations induced by meteorology. Significant variations were observed in indoor PM_10_ concentrations during classroom occupancy (up to 230 µg m^−3^) vs. non-occupancy (up to 19 µg m^−3^) days, finding levels higher than outdoor ones. This was attributed to the scholarly activities in the classroom. Conversely, PNC and eBC concentrations only increased when the windows of the classroom were open. These findings have helped to establish practical recommendations and measures for improving the IAQ in this school and those of similar characteristics.

## 1. Introduction

Air pollution is a serious matter of concern in big or densely populated cities. Generally, road traffic is a dominant source of air pollutants like NO_x_, ultrafine, and black carbon (BC) particles [1]. Although not all these pollutants are regulated, their health effects are clearly recognized [2,3,4], so they must be monitored. However, indoor pollutants are not sufficiently characterized in comparison with outdoor ones. Except for labor or specific industrial environments, many countries have no established regulations for indoor air quality, which makes indoor air pollutant behavior much less known [5].

Children are one of the most vulnerable population groups to indoor air pollution as they are still in a developing stage [6,7,8]. In addition, their lung surface area per body weight is larger and they have more frequent mouth breathing for physical activity compared to adults [9,10]. Thus, they are exposed to more air pollution than adults [11]. Consequently, a number of respiratory pathologies, including asthma, allergies, or airway inflammation, have been identified as associated with children’s exposure to indoor air pollutants [12,13,14,15,16]. In addition to other indoor environments, such as at home, children spend a large amount of their day at school [12].

In schools, poor indoor air quality (IAQ) situations have already been recognized that can affect children’s health, causing or contributing to acute and chronic health problems [15]. An association between indoor air pollutants and frequent respiratory problems has been found [12]. Rhinoconjunctivitis has been associated with high levels of formaldehyde, ethylbenzene, and xylenes in classrooms, and even with a high PM_2.5_ concentration [17,18]. Asthma exacerbation has also been related to indoor PM_2.5_ concentration in school [17]. Dermal symptoms in children such as skin allergy have been associated with exposure to PM_10_ [19] and a frequent occurrence of dermal irritation with high aromatic hydrocarbon concentrations [18]. There is also a strong influence of indoor air quality in schools on children’s well-being [15,20]. Some studies have found an association between poor IAQ and the loss of students’ attention in the classroom and their academic achievement [21,22,23,24], also with an impact on learning progression [23,25] or with effects on neurobehavioral function in children [26].

All of the above-mentioned points have led to experimental studies on indoor air quality being carried out in different parts of the world [27] and in different types of environments where the building is located [28]. Despite evidence that continues to emerge showing that a poor IAQ can cause health problems, most countries do not have yet control policies, among them Spain. Moreover, not all the pollutants identified inside the schools are regulated, like BC and particle number concentration. Fine particles (especially <100 nm) are more numerous than large ones but do not contribute much to PM mass. Due to their small size, these particles have a greater ability to enter the body through breathing. Thus, monitoring only PM mass could be inefficient in tackling their health effects. In this regard, the World Health Organization (WHO) has set, for the first time, specific indoor and outdoor air quality guidelines for the number concentration of particles larger than 10 nm (>10,000 particles cm^−3^/24 h or 20,000 particles cm^−3^/1 h). In addition, WHO has also reviewed some levels of air pollutants to protect human health such as PM_2.5_ (15 µg m^−3^ 24 h mean), PM_10_ (45 µg m^−3^ 24 h mean), ozone (100 µg m^−3^ 8 h mean), nitrogen dioxide (25 µg m^−3^ 24 h mean), sulfur dioxide (40 µg m^−3^ 24 h mean), or carbon monoxide (4 mg m^−3^ 24 h mean) [4]. To protect human health, it is essential to know the pollutant concentrations and identify the outdoor and indoor sources, their temporal variability, and sinks to assess the children’s exposure to air pollutants and the associated health risks.

In an important number of cities, schools are located within the urban environments and in some cases near high-traffic urban roads with bus stops. Thus, many IAQ studies in different cities around the world like Barcelona (Spain) [29], Lisbon (Portugal) [30], Wellington (New Zealand) [31], Hangzhou (China) [32], Cassino (Italy) [15], La Rochelle (France) [33], and Antwerp (Belgium) [34], among others, are focused on this type of school environment. Their main finding is that traffic emissions can be considered the main air pollutant source to which children are exposed [35].

Infiltration of pollutants from the outdoor air is one of the main mechanisms that affect IAQ in schools [36]. This process is complex and highly variable. Time-related factors, such as emissions from outdoor sources [28] and meteorological conditions [37], influence the spatial and temporal patterns of air pollutants and can strongly affect their accumulation at the local scale. In this regard, a large number of studies have found significant associations between these factors and infiltration processes. Thus, traffic-related pollutants have been identified in school indoor environments, including BC, particulate matter (PM), ultrafine particle number concentration (PNC), or PAHs [29,31,32,34,38,39,40,41]. In addition, meteorological conditions play a fundamental role in the exchange of air. Different studies have demonstrated the relationship between meteorology and indoor/outdoor (I/O) levels. Chithra and Nagendra [42] or Elbayoumi et al. [43] found an association between I/O ratios and wind speed and direction, while [44] observed this association only with wind speed. In this regard, delay times found in the literature ranged from ~1 [29,45] to several hours [46,47]. However, Chan [37] identified that temperature, humidity, and solar irradiation play a vital role in the variation of the I/O pollutant concentrations and exchange. In this context, building characteristics (sealing the building, age, number of windows or doors) and occupancy-related activities, such as ventilation frequency, duration, or when it occurs, can also have a large effect on the air exchange rates from occupied spaces [48].

But, in many cases, indoor sources are negligible. In this regard, common pollutants in indoor air such as PM_x_ (PM_10_ and PM_2.5_) from textiles [49,50], using chalk dust [51,52], or particulate resuspension linked to the activities of occupants [43,53,54] can be expected in classrooms, strongly influencing IAQ [55], in the same way that indoor volatile organic compounds (VOCs) originate from cleaning products [50,56], furnishings, or building materials [57]. Thus, specific air pollutants can usually present higher levels indoors than outdoors during the occupancy in schools [34,40,58,59,60,61].

All the aforementioned information highlights that schools are complex microenvironments with different factors, especially those related to meteorology and pollution sources, which can contribute to the deterioration of IAQ. This feature, together with the lack of pollutant standards for indoor environments, both for parameters and their levels, calls for further and more detailed studies to provide environmentally responsible measures. Thus, an intensive measurement campaign (15 November to 22 December 2017) was carried out for the first time in Madrid to characterize the children’s exposure to air pollutants in three school environments with different characteristics. As an essential part of this campaign, this work presents the study of the most affected school by traffic emissions (hourly equivalent black carbon (eBC) concentrations up to 14.6 µg m^−3^) [62,63]. For this purpose, a cold period representing the most likely adverse pollution episodes in the region was selected. Simultaneous indoor/outdoor measurements of PM-related pollutants (PM_10_, PM_2.5_ and PM_1_, PNC, and eBC), were carried out at three different measure points of the school. Complementary data of trace gases (SO_2_, NO, NO_2_, and O_3_) and meteorology were measured and included in the analysis. The next three objectives were defined in this investigation: (i) to characterize the outdoor air pollutants in the school’s surroundings, (ii) to evaluate the influence of outdoor pollutants and meteorological parameters on indoor concentrations, and (iii) to assess the impact of different classroom activities on IAQ.

### 1.1. Experimental Site and Measurements

Madrid city, the capital of Spain, including its metropolitan area, is the most populous urban concentration of the country, with over 6 million inhabitants. Heavy industry is absent, so road traffic and home heating during the cold period are the main air pollution sources of the city [64].

The climate in this region can be classified as the Continental–Mediterranean type, influenced by urban features [65]. The Madrid basin is surrounded by mountainous systems, the highest in the north–northwest region. Thus, local wind circulation is conditioned by the mountain breezes.

The measurement campaign was carried out a month before the beginning of winter, between 22 November 2017 and 21 December 2017, being part of the cold period in Madrid. The selection of this study period is because meteorology and geographical factors in the cold period result in high pollution levels in the city [66] that could have significant consequences for population health [67], and temperatures are lower than in summer, resulting in children spending more time indoor and with poor ventilation to reduce heat loss. The formation of persistent anticyclonic systems over the Iberian Peninsula with strong surface temperature inversions on a local scale is frequent in the area during this period [68], enhancing atmospheric stability. The low wind speed and the lack of precipitations trap urban pollutants within a narrow surface layer by poor ventilation. These situations can be interrupted by the passage of cold fronts from the North Atlantic that are associated with precipitation events and high wind speed, leading to cleaner conditions. Additionally, Saharan dust outbreaks, although more frequent in summer and spring, can also reach Madrid in the cold period [69]. These Saharan air masses are usually characterized by warm, dusty, and dry. This results in high PM_x_ levels and enhancing atmospheric stability conditions due to (i) a decrease in insolation at surface levels and, accordingly, the convective mixing due to the dust layers, and (ii) an increase in the thermal inversions, promoting an increase in local pollutant concentrations on the surface [70,71].

Despite measures taken by authorities and that air quality action plans are activated during pollution episodic conditions [72], high levels of PM_x_ and even exceedances of several pollutant standards (i.e., NO_2_ hourly limit value of 200 µg·m^−3^) are frequently recorded under these unfavorable situations in many stations of the Madrid air quality monitoring network [73,74].

**Figure 1:**
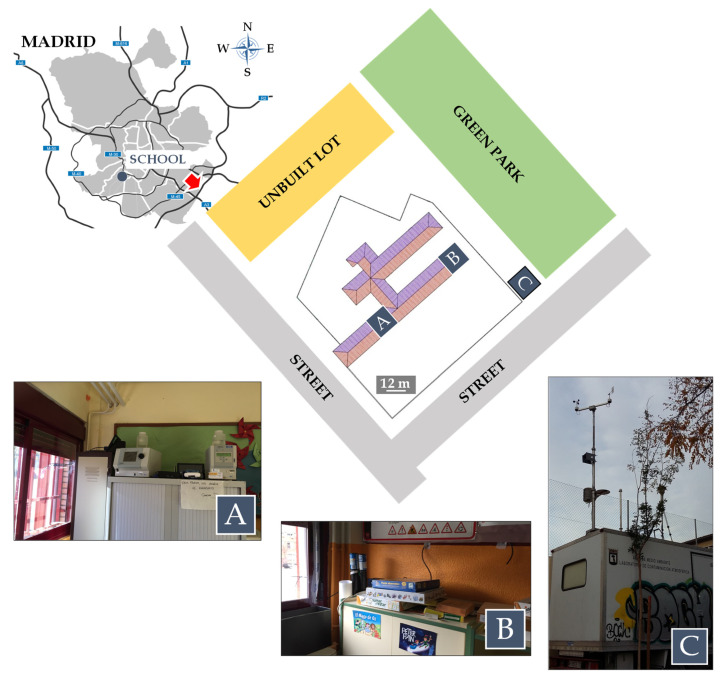
The blue circle in the map shows the location of the school selected for this study and the red arrow indicates its building scheme (**top**), identifying the sampling point locations (**bottom**): classroom A (**A**), classroom B (**B**), and external checkpoint outside the school grounds (**C**).

This study was performed in one of the most affected areas by traffic emissions, a primary school located in the southwest city downtown in an urban residential zone, adjacent to an unbuilt lot, a green park, and two busy-traffic streets (Figure 1, top panel). Thus, the school surroundings are strongly influenced by traffic emissions, with the main and back entrances being ~30 m from both streets. These and adjacent streets, as in the whole district, are carrying moderate to heavy traffic during the mornings and evenings, coinciding with the arrival and departure times of scholars. Unfortunately, no traffic counters are available for these streets, recording the closest ones (~300 m) from 41 to 501 on the daily average of each 15 min interval during the campaign (https://datos.madrid.es/portal/site/egob/ (accessed on 28 November 2023)).

Three different sampling points were considered for this study: two classrooms (A and B) and an external checkpoint (C), located by the fence outside the school grounds, in a mobile air quality monitoring unit provided by the Madrid municipal authorities (Figure 1, bottom panel). The classrooms were located on the first floor of a three-story building with windows oriented to a closed schoolyard. Thus, they were selected not only as being representative of the occupancy in the building but also for analyzing the influence of traffic emissions on IAQ in detail, while sampling point C, close to the street, was selected as representative of the outdoor air quality levels. The distance between the three measuring points was between 30 and 60 m above ground level (a.g.l.), and all of them were located ~3–4 m above street level. Both classrooms are usually occupied by students during school hours and days. In addition, the case of classroom A was naturally ventilated by opening windows on 27 (09:30–10:15 and 13:00–13:44 UTC), 28 (10:45–13:05 UTC), 29 (11:30–13:00 UTC) and 30 (11:30–12:50 UTC) November, and 1 (09:00–10:24 and 12:20–13:05 UTC), 4 (07:55 and 10:45–11:30 and 12:20–12:50 UTC), 05 (07:50–08:55 and 13:23–13:51 UTC), 13 (10:30–11:00 UTC), 14 (09:30–10:10 and 11:30–13:50 UTC), 15 (09:10–09:31 UTC), 18 (10:50–11:30 and 12:15–13:40 UTC), and 20 (08:02–09:07 and 12:20–12:50 UTC) December.

Simultaneous continuous measurements of different constituents and co-pollutants of particulate matter (PM), referred to here as PM-related pollutants, were obtained at the three sampling points. Ultrafine particle number concentration (PNC) was measured with Condensation Particle Counters (CPC) TSI models 3775 and 3772 (TSI Inc., Aachen, Germany), measuring particles greater than a size cut-off of 4 and 10 nm, respectively. BC data were obtained, derived from the optical absorption method by microAeth^®^ model AE51 (Aethlabs, San Francisco, CA, USA), so it is referred to as equivalent black carbon (eBC) [75]. Finally, a Grimm portable Laser Aerosol Spectrometer-Model MiniLAS 11-R (Grimm Technologies, Ainring, Germany) was used to measure PM_10_, PM_2.5_, and PM_1_ mass concentrations. This instrumentation was distributed in three different points, as indicated in Table 1. Indoor and outdoor instruments were installed inside classrooms of the primary school. The outdoor ambient air sampling line was taken out through one of the windows of each classroom (A and B), and once installed, the windows were sealed to obtain representative indoor conditions of closed windows. In some periods, PNC obtained by the CPC model 3772, exceeded the maximum value accepted by the instrument (10,000 cm^−3^), and exceeding, in some hours, the value recommended by the WHO (20,000 particles cm^−3^).

All instruments were verified and intercompared before starting the measurements campaign and configured to record data every minute. The calibration of the CPCs was checked during the Spanish Network of Environmental DMAs (REDMAAS) intercomparisons [76]. AE51 microethalometers (Aethlabs, San Francisco, CA, USA) were intercomparated with a continuous Aethalometer (Magee Scientific Aethalometer model AE33, Aerosol d.o.o., Ljubljana, Slovenia) belonging to the ACTRIS Network and followed its standard procedures [77], obtaining a correction factor AE51 = ~1.2 × AE33 (R^2^ = 0.74–0.77). Data obtained by Grimm’s portables were corrected using the data from TEOM installed at the CIEMAT site as a reference [78] and which is periodically calibrated by the manufacturer. The calibration curves for the outdoor GRIMM were y = 0.834x + 1.6 and y = 0.849x + 1.2 and for the indoor GRIMM of y = 0.820x + 1.1 and y = 0.848x + 0.4 for PM_10_ and PM_2.5_, respectively.

Outdoor measurements were carried out at the three measuring points, as follows: (i) eBC, PNC, and PM_x_ were measured from classroom A, (ii) eBC was measured from classroom B, and (iii) eBC and PNC were obtained from the external checkpoint C. Further air quality parameters (SO_2_, NO, NO_2_, O_3_, and PM_10_) were monitored by the unit at the external checkpoint (Table 1). Data from the Madrid Air Quality Monitoring network were not used. The closest station to the study area (~1.2 km away), the Fernandéz-Ladreda traffic station, is a hot spot in Madrid, recording the highest NO_2_ concentrations in Madrid City and the whole Madrid region (Madrid City Council [79] and previous annual air quality assessment reports). Thus, this information in terms of pollution levels is not representative of the study area, with the one obtained from the external checkpoint being used as representative of the local conditions in the school environment. In addition, simultaneous indoor measurements were also carried out in both classrooms, A and B. Figure S1 shows the indoor/outdoor time series obtained in these classrooms during the sampling period. Data gaps were due to instrument maintenance.

Meteorological data provided by a high meteorological tower (52 m.a.g.l) located in a suburban area, at the CIEMAT site [78], ~8 km from the measurement site, were used for the analysis. The meteorological tower is situated in the area northwest of Madrid, a few kilometers from the city center, being bounded by natural areas on three sides (southwest, northwest, and northeast) and ~3 km away from built areas. Thus, meteorological data are not affected by local buildings or shielding and are representative of the general atmospheric situation in the urban area. Atmospheric pressure (AP) was measured at ground level, temperature (lower T) and relative humidity (RH) at 31 m.a.g.l., and temperature at a second height (upper T) and wind speed (WS) and direction (WD) at 52 m.a.g.l. The temperature difference between the two levels (surface temperature lower than registered at height) was used to identify surface inversion periods and, thus, atmospheric stability conditions. All meteorological data were provided each 10 min.

Ancillary meteorological products were used in this study, supporting the meteorological analysis. Synoptic-scale air mass changes over the Iberian Peninsula affecting the Madrid area were analyzed through the use of the surface synoptic charts provided by the National Meteorology Agency (AEMET). Also, the Dust Regional Atmospheric Model (DREAM: https://www.bsc.es/projects/earthscience/visor/sub_fc8.php?type=dld&dom=med8 (accessed on 16 February 2024)) was used to identify Saharan dust outbreaks that took place during the experimental period.

The coevolution between the meteorological data and products corresponding to the study period with air quality information obtained at the external checkpoint in the school made it possible to identify the different meteorological conditions, conditions of atmospheric stability, instability, and intermediate. For this purpose, the information provided by previous research studies related to meteorological aspects relative to the relationship between the synoptic circulation and mixed-layer evolution and seasonal meteorology in Madrid was also considered [66,68,69].

A daily activity sheet was used to collect information on the study occupant’s activity pattern in classroom A, including children and teacher occupancy, opening/closing windows, or a combination of them. Based on this information, four occupational activities were differentiated: (i) non-occupancy period with closed windows, i.e., air infiltration from outdoor, (ii) non-occupancy period and classroom ventilated naturally with open windows, (iii) occupancy period with closed windows, and (iv) occupancy period with classroom ventilated naturally (open windows). In the classrooms, air circulation from indoor to outdoor is not forced by any mechanical system. The air concentration gradient governs the diffusion entry rate indoor. Thus, in the absence of indoor sources, as occurred in this study, the entry rate via this mechanism will be higher, resulting in a gradient from outdoor to indoor. Consequently, the pollutant interchange from indoor to outdoor can be negligible.

Pollutants measured were analyzed using the four daily periods established by Salvador et al. [80] based on typical pollution conditions in Madrid: 00:00–04:00 UTC (night period as a background pollution scenario), 05:00–09:00 UTC (morning period, including morning rush hour peak), 11:00–15:00 UTC (midday period), and 17:00–21:00 UTC (intermediate period, including the afternoon rush hour peak).

Data and meteorological information herein are provided in UTC time (local time: 1 h) and averaged over 10 min for comparison purposes.

### 1.2. Data Analysis

Descriptive analyses were used to evaluate the influence of outdoor pollutants and meteorological characteristics on indoor concentrations. Thus, indoor/outdoor (I/O) ratios were calculated from the 10 min database obtained during the campaign, being used to estimate the infiltration factor. As occurred in classrooms A and B, in the absence of indoor activity, without indoor pollutant emissions and under steady-state indoor conditions, the infiltration factor (*F*_*in*_) can be formulated as an I/O ratio [81]. As pollutant data are predominantly log-normally distributed, the geometric mean is the main statistical parameter used to present data. I/O ratios < 1.0 indicated that contributions from indoor pollutants were less than from outdoor ones, while I/O ratios > 1 showed the dominance of indoor contribution.

The I/O ratios and meteorology were used as a database for the analysis of variance (ANOVA). The I/O ratio values were considered as dependent variables, while meteorology (atmospheric stability and instability and wind speed) factors were chosen as independent (or predictive) variables. One-way ANOVA calculated I/O means and variances between and within the different categories. *p*-values < 0.05 were considered significant. All data were analyzed using R 4.3.0 statistical software^®^.

## 2. Results and Discussion

### 2.1. Overview: Meteorology and Local Air Quality

**Figure 2:**
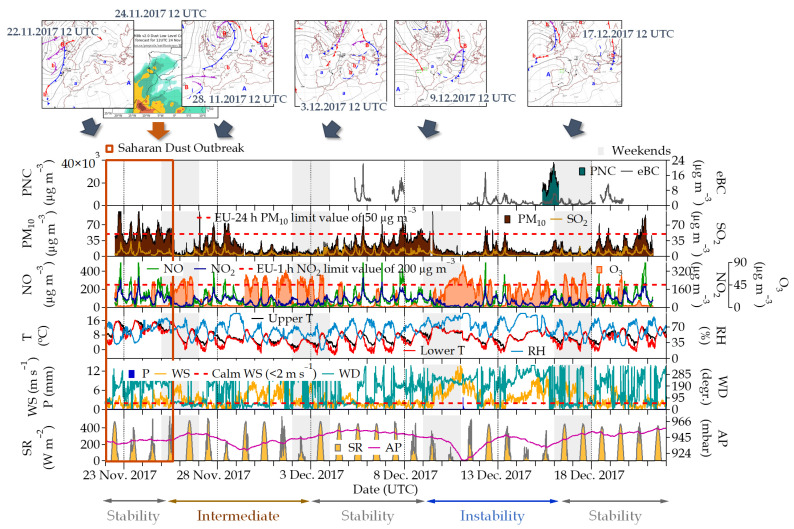
Temporal evolution of PNC, eBC, PM_10_, and trace gases (SO_2_, NO, NO_2_, and O_3_) obtained at the external checkpoint and meteorology information (upper and lower temperature (upper and lower T, respectively), relative humidity (RH), wind speed and direction (WS and WD, respectively), precipitation (P), solar radiation (SR), and atmospheric pressure (AP)) obtained at the CIEMAT site during the period of monitoring. In the graph, “Stability” refers to atmospheric stability periods, “Intermediate” to intermediate atmospheric periods, and “Instability” to atmospheric instability periods. The main surface pressure analysis maps and dust load maps describing the different atmospheric periods identified in this study are incorporated on top of the figure.

Meteorological information together with the pollutants measured at the external checkpoint were used as a reference to identify different atmospheric periods during the measurements campaign (Figure 2). Stability conditions alternated with high dispersion ones in different periods that can be described as follows:

Atmospheric stability periods: Three atmospheric stability periods were identified during the measurement period: (i) 22–25 November 2017, (ii) 3–9 December 2017, and (iii) 17–22 December 2017. Prevailing calm conditions (wind speed < 2 m s^−1^ in height), surface temperature inversions, and high atmospheric pressure were common in these periods, reducing the pollutant dispersion. High ambient PM_10_ hourly levels were found, exceeding the 50 µg m^−3^ European Union (EU) daily limit value on some occasions. In addition, SO_2_, NO, and NO_2_ concentrations significantly increased, coinciding with the increase in PM_10_. In the same way, SO_2_ concentrations were slightly higher than in the rest of the atmospheric situations identified, ~10 µg m^−3^ on average (Table S1). NO_2_ hourly concentrations reached the EU limit of 200 µgm^−3^, with values of 217 µg m^−3^ (22 November 2017, at 19:00 UTC) and 218 µg m^−3^ (23 November 2017, at 18:00 UTC). This resulted in the activation of the protocols for air pollution control due to high NO_2_ levels by the Council of Madrid City (https://www.madrid.es/portales/munimadrid/es/ContenidosBasicos/2017-Episodios-de-contaminacion-Protocolo-dioxido-de-nitrogeno-/?vgnextfmt=default&vgnextoid=d17d0d81f9652710VgnVCM2000001f4a900aRCRD&vgnextchannel=a1cc8f348af1c310VgnVCM2000000c205a0aRCRD (accessed on 18 September 2023)) between 15 and 24 November 2017 and 5 and 8 December 2017. These protocols have been proven to be effective in their final stage with a significant effect on NO_2_ maximum concentrations, although further studies must be carried out to estimate more precisely the effect of the measures taken and to assess potential trade-offs [74]. O_3_ concentrations were low, typical of this period of the year, and especially for emission areas where O_3_ is reduced by titration with NO. The DREAM model indicates a Saharan dust outbreak between 22 and 25 November 2017. The daily average concentration of PM_10_ varied between 64 (22 November) and 34 µg m^−3^ (25 November), finding during these days the highest PM_10_ daily concentration of the period study (Figure 2).

Atmospheric instability periods: A period (9–17 December 2017) of atmospheric instability conditions occurred during the experimental campaign. This was characterized by the influence of a low-pressure system (Figure 2) and higher wind speeds, up to 13.6 m s^−1^. A precipitation event was registered on 11 December 2017, accumulating 10.6 mm (Table S1). These high dispersion conditions allowed a greater dilution of pollutants, which, along with wet deposition, reduced the PM_10_ concentrations and traffic emission gases in the surface layer. Thus, the daily average concentrations of the different pollutants measured were lower than those for the stability periods; in this case between 7 and 21, 2 and 6, 2 and 70, and 14 and 62 µg m^−3^ for PM_10_, SO_2_, NO, and NO_2_, respectively. In this period, the O_3_ concentration was higher, as shown in Figure 2, given that NO levels were low during the period and O_3_–NO titration did not occur.

Intermediate Atmospheric periods: Finally, a period of intermediate synoptic conditions was identified between 25 November and 3 December 2017, with different ventilation scenarios. In this last situation, we observed sunny days alternating with cloudy days with some precipitation (2.2 mm, Table S1). During this period, wind speed ranged from 0.1 to 9.1 m s^−1^, finding hourly values for PM_10_, SO_2_, NO, and NO_2_ between 2 and 71, 2 and 26, 1 and 343, and 8 and 160 µg m^−3^, respectively.

### 2.2. Outdoor Air Pollutants in the School’s Surroundings

**Figure 3:**
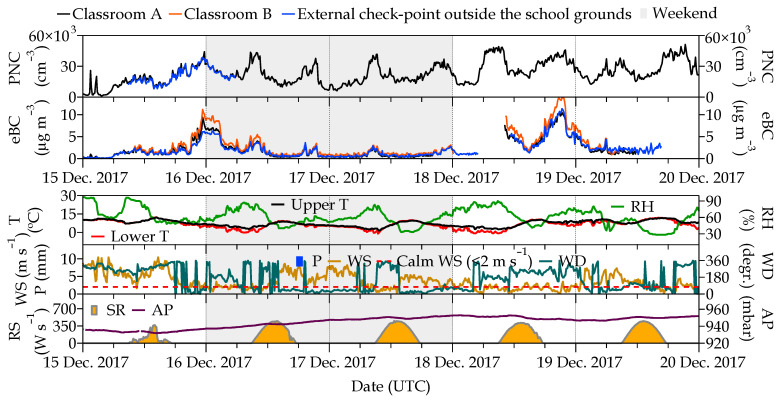
**Upper panel**: Simultaneous time series of outdoor PNC and eBC measured at the three sampling points. **Lower panel**: Temporal variation of meteorological parameters (upper and lower temperature (upper and lower T, respectively), relative humidity (RH), wind speed and direction (WS and WD, respectively), precipitation (P), solar radiation (SR), and atmospheric pressure (AP)) obtained at CIEMAT site during the period 15–19 December 2017.

The outdoor air pollutants in the school’s surroundings were analyzed over 5 days which are representative of the meteorological variability and emission sources (weekend and work days) found in this study. Outdoor PNC and eBC concentrations measured simultaneously at the three different points exhibited the same temporal pattern (Figure 3).

Outdoor PNC levels varied from 1176 cm^−3^ (classroom A) to 38,330 cm^−3^ (external checkpoint) and from 0 µg m^−3^ (external checkpoint) to 13.8 µg m^−3^ (classroom B) for eBC. PNC concentrations were quite similar at the three sampling points, exceeding hourly (20,000 particles cm^−3^) and daily (>10,000 particles cm^−3^) levels recommended by the WHO, as well as eBC levels. They also showed a similar trend, although slight differences in eBC concentration were found between the points in classrooms A and B when these concentrations reached the maximum values. In general, the behavior of PNC and eBC concentrations seemed to indicate a rather good mixing of the airmass.

At this point, it must be highlighted that both pollutants exhibited two peaks on most days: one of them in the morning (07:00–09:00 UTC) and the other in the evening (20:00–21:00 UTC), which can be associated with rush hour traffic emissions. This same pattern was also observed in outdoor NO_x_ and PM_10_ levels (Figure 2), further confirming the traffic emissions origin of PNC and eBC. In addition, higher concentrations on workdays than on weekends indicated that PNC and eBC had a strong link to traffic emissions. This relationship was especially stronger for eBC than for PNC, showing a clear traffic origin of that pollutant. Therefore, PNC and eBC could be considered as tracers for children’s exposure assessment to pollution from outdoor sources, in this case, traffic. Unlike other studies, such as Kumar et al. [35], pollutant concentration peaks associated with the influence of traffic emissions and particle resuspension during the arrival and departure to the school were not identified in the present study. This suggests a substantial overlap between the local emission sources and urban-level emissions.

### 2.3. Indoor Air Quality and Impact of Outdoor Air by Infiltration

Relationships between indoor/outdoor (I/O) concentrations of PNC, PM_x_ (PM_10_, PM_2.5_, and PM_1_) and eBC were determined in the absence of any human activity in both classrooms.

**Figure 4:**
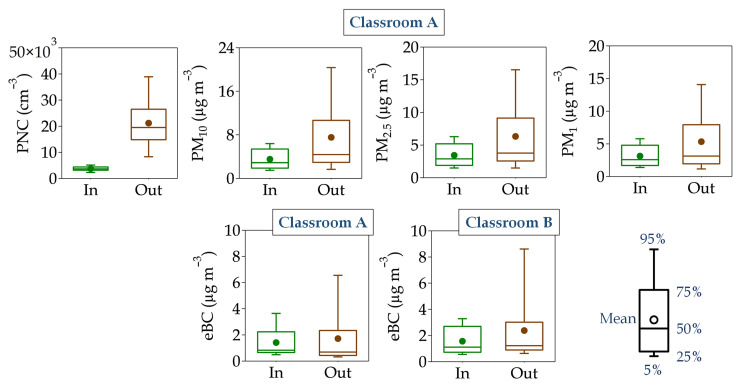
Box plot of 10 min indoor and outdoor concentrations for all pollutants (PNC, PM_10_, PM_2.5_, PM_1_, and eBC) measured in the two classrooms (A and B) during a non-occupancy (weekend) period (from 15 December 2017, at 21:10 UTC to 17 December 2017, at 20:10 UTC) with all available data. In the graph, the prefix “Out” refers to outdoor, whereas “In” indicates indoor measurements.

Indoor levels of PNC, PM_x_, and eBC were in the ranges of 1956 to 6109 cm^−3^, 1 to 19 µg m^−3^, and 0.4 to 1.5 µg m^−3^, respectively, always lower than the corresponding outdoor levels that varied from 5975 to 47,180 cm^−3^, 1 to 119 µg m^−3^, and 0.2 to 3.7 µg m^−3^, respectively, in the same period. The measured indoor PM_x_ values confirmed that there are no indoor sources of particles and that in the absence of classroom activity, PM_1_ was the main contributor to PM_2.5_ and PM_10_. In this regard, and for more detailed analysis, 10 min concentrations of these indoor/outdoor pollutants for the two most complete non-occupation periods of the study are presented in Figure 4. The highest indoor and outdoor air pollution levels were associated with atmospheric stability vs. instability periods (Table S2). Comparing the observed values found here with those reported in the literature for urban areas, a similar trend was found, i.e., indoor pollutants were lower than those in ambient air; Portela et al. [82] found values for PNC and eBC of 2.44 × 10^4^ and 4.76 × 10^4^ cm^−3^ and 3.07 and 5.53 µg m^−3^ in indoor and outdoor air, respectively, in the State of Rio Grande do Sul (Brazil). Similar PNC concentrations were observed in Cassino (Italy) [83]. At this site, the indoor PNC values varied between 2.04 × 10^4^ and 3.49 × 10^4^ cm^−3^ for indoor environments and between 2.77 × 10^4^ and 4.72 × 10^4^ cm^−3^ for outdoor ones [84]. However, eBC levels were found to be higher, indoor eBC levels ranged between 1.9 and 13.9 µg m^−3^, and outdoor levels from 3.2 to 16.3 µg m^−3^ [83]. Hassanvand et al. [85] reported PM_2.5_ and PM_1_ levels of 19.0 and 33.6 µg m^−3^ and 11.0 and 25.4 µg m^−3^ for indoor and outdoor air, respectively, in schools in Tehran (Iran). In Barcelona (Spain), Viana et al. [86] reported average values for PM_10_ lower than 24.3 and 26.0 µg m^−3^ in indoor and outdoor school environments, respectively. Subsequently, Reche et al. [29] found values for eBC of 1.3 and 1.4 µg m^−3^ and for PNC of 1.6 × 10^4^ and 2.3 × 10^4^ cm^−3^. The different concentrations among these studies may be attributed to factors such as meteorological conditions and ambient urban pollution levels, especially from traffic, as well as the different influence of local sources in every case.

**Figure 5:**
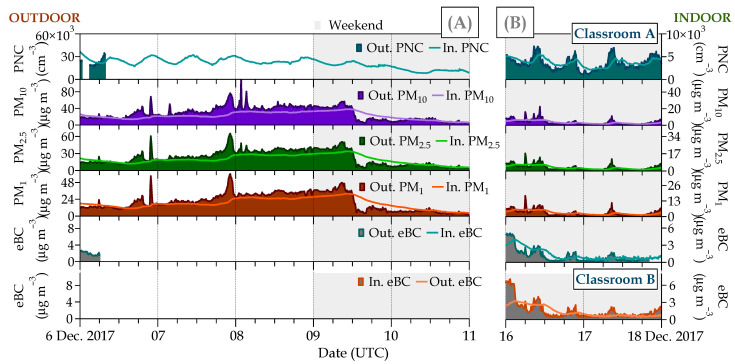
Simultaneous time series of indoor/outdoor (I/O) pollutants (PNC, PM_10_, PM_2.5_, PM_1_, and eBC) measured at classrooms A and B during two non-occupancy periods (weekends and vacation), from (**A**) 6 to 11 December 2017 (left panel) and (**B**) 16 to 18 December 2017 (right panel), with stable and ventilated atmospheric conditions, respectively. In the graph, the prefix “Out” refers to outdoor, whereas “In” indicates indoor measurements.

The indoor eBC levels showed a similar pattern in the two classrooms, so they are affected by the same (external) sources. Variations in wind speed and direction alter the atmospheric conditions of turbulence, thereby outdoor pollutants rapidly can change in space and time. These changes were also observed in the indoor levels, confirming infiltration processes, albeit they were gradual and delayed. Thus, indoor peaks correlated with outdoor peaks but appeared to be shifted between 0.5 and 2 h (Figure 5). These delay times are within those found in previous studies on IAQ in schools. Reche et al. [29] and Xu et al. [45] estimated delay times of ~1 h, while Hu et al. [46] and Tippayawong and Khuntong [47] identified delay times from 1 to a few hours. Here, wind speed appeared to be an important factor in modulating the infiltration of outdoor pollutants into indoor air [87]. The higher the wind speed, the lower the delay times that were observed, i.e., they were negatively correlated, finding determination coefficients (R^2^) from 0.4 to 0.8 between both parameters. This could suggest that atmospheric dispersion favored the infiltration of outdoor pollutants into classrooms (Table S2). Other studies, like Kearney et al. [88] or Wan et al. [89], have also found a similar association: a higher wind speed leads to higher I/O air exchange.

To assess the influence of atmospheric stability, Table 2 shows I/O ratios of the pollutants monitored in the two classrooms in the absence of students during two periods with different atmospheric conditions, one of them of atmospheric stability (from 6 (00:00 UTC) to 9 December 2017 (11:00 UTC)) and the other of instability (from 16 (00:00 UTC) to 17 December 2017 (21:00 UTC)). Statistically significant differences between the two periods according to changes in wind speed are observed.

The highest I/O ratios were found under atmospheric instability conditions (Table 2). Except for PNC and PM_10_, I/O ratios higher than 1 were identified for all pollutants, meaning that indoor concentrations were higher than outdoor ones during certain periods of the day. This mainly occurred at midday (I/O ratios up to 2 for eBC) when WS reached more than 3 m s^−1^, after a calm period (morning). These findings can be associated with rapid changes produced by wind in outdoor concentrations than indoor, whereas indoor ventilation is very low, resulting in higher I/O ratios. On average, the highest I/O ratios were found for PM_2.5_ and PM_1_ (0.7) and eBC (1.2). Concerning the eBC, in addition to the rapid changes in outdoor concentrations, this finding may be related to the low chemical reactivity of eBC with other species [90] or be the result of its low diffusivity capacity. In this last regard, the particle sizes of eBC are below 300 nm [91] where the Brownian diffusion is more effective as a transport mechanism. Furthermore, processes like coagulation under high ventilation conditions are relatively unimportant for this range of particle sizes for which the particle mass is low [92], as occurs in this study. A comparison of the values of eBC I/O ratios between the two classrooms showed that the air change rate was slightly lower in classroom B. This can be due to the higher airtightness of this classroom. The eBC is the main component of the smallest particles and a Category I Carcinogen according to the WHO [93]. This is linked to children inhaling a higher concentration of fine particles than adults, which may result in a high potential risk to their health [94].

Regarding PM_x_ mass concentration, some studies showed a dependency between I/O ratios and the particle concentration and its size, being higher for larger particle sizes [81,95]. However, in this study, outdoor ambient PM_x_, especially PM_10_ and PM_2.5_, had less impact on indoor concentrations, unlike the other studies [85,96], where outdoor PM_x_ concentrations were higher (see PM I/O rates in Table 2). In fact, PM_1_ was the largest contributor (95%) to PM_10_ levels during the non-occupancy period (Table S2). Particles larger than 2.5 µm often have large contributions from outdoor sources different than direct traffic combustion emissions, such as road traffic resuspension, street works, demolition, or African dust outbreaks [97,98].

The lowest I/O ratios (average of 0.2) during the non-occupancy period were found for PNC. Here, this result could be influenced by the particle penetration efficiency from the outdoor environment and the particle deposition rate on indoor surfaces in different particle sizes [99]. In addition, as previously discussed, local traffic emissions impacted the concentrations of outdoor particles and, consequently, in the absence of indoor sources, they had a clear origin from outdoor air [36].

I/O ratios higher than 1 were not found during the atmospheric stability situation, although they were slightly higher at night for all pollutants. This could be associated with surface pollutant accumulation by reduction in the boundary layer thickness and the inhibition of dispersion processes under these conditions.

These findings were statistically verified. Obtained differences are significative according to ANOVA results with *p*-values consistent with high significance levels, between <2 × 10^−16^ and 2.14 × 10^−6^. It means that meteorological conditions influenced the IAQ, confirming the results found on their influence on infiltration processes in the classrooms. Complete information about the ANOVA results can be found in Table S4.

Other studies have analyzed the I/O ratios during non-occupancy periods. Park et al. [100] reported I/O ratios of 0.68 for BC and 0.53 for PM_2.5_ in an elementary school in Gyeonggi-do Province (Republic of Korea). I/O ratios of 0.55 and 0.25 were obtained by [101] for soot and PM_2.5_ in schools in Stockholm (Sweden). Both studies identified a similar trend to those found in this study (eBC I/O ratios > PM_x_ I/O ratios). A mean I/O ratio of 0.77 ± 0.04 for PM_2.5_, between 0.34 and 0.81 for ultrafine particles (15–30 nm), and a higher I/O ratio (0.90–0.93) for accumulation mode particles (102–737 nm) were obtained in schools of Brisbane (Australia) [102]. Chen et al. [103] found I/O PM (0.3–10 µm) ratios between 0.61 and 0.95 in classrooms at five primary schools in different regions of Singapore. Lower I/O ratios were identified for empty classrooms with mechanical ventilation [104] in an elementary school in Salt Lake City (UT, USA). Discrepancies among infiltration factors were influenced by the microenvironment and building characteristics and, consequently, by the air exchange rate. Regardless of I/O discrepancies found in these studies, mostly, the differences found among them for traffic-related pollutants (PNC, PM_x_, and eBC) could be related to particle diameters [105], as this study suggests.

### 2.4. Effects of Occupancy on the Indoor Air Quality

**Figure 6:**
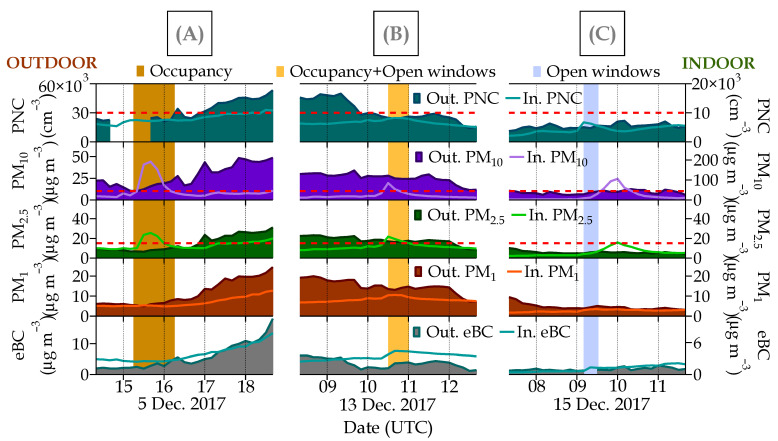
Simultaneous time series of indoor/outdoor (I/O) pollutants (PNC, PM_10_, PM_2.5_, PM_1_, and eBC) measured at classroom A. Examples of the three different activities in the classroom considered in this study are (**A**) an occupancy period, (**B**) a window-open period during occupancy, and (**C**) an open-window period without occupancy. The red dotted line in PNC represents the optimal detection range of the indoor CPC (0–1 × 10^4^ cm^−3^), in PM_10_ this dotted line represents the WHO 24 h standard of 45 µg m^−3^, and in PM_2.5_ it represents the WHO 24 h standard of 15 µg m^−3^ for indoor ambient air levels. In the graph, the prefix “Out” refers to outdoor, whereas “In” indicates indoor measurements.

Different situations, such as occupancy, opening windows, and the combination of both, were recorded in classroom A during the sampling period. Consequently, indoor pollutant levels fluctuated from day to day depending on the classroom conditions, as shown in Figure 6.

The observed data highlighted the strong influence of occupancy on PM_x_ indoor concentrations. Mean (sd) concentrations of PM_10_, PM_2.5_, and PM_1_ during the occupancy periods were 19(28), 7(5), and 4(2) µg m^−3^, respectively (Table S3). Peak concentrations were observed simultaneously in the three size fractions (PM_10_, PM_2.5_, and PM_1_), with a maximum concentration of 230, 41, and 11 µg m^−3^, respectively, observed on 18 December at 11:20 UTC. These high levels were associated with particle resuspension in the air that occurred during children’s workout activities in the classroom. Note that chalk is not used in this classroom, aimed at performing psychomotor activities such as sitting, picking up toys, or moving around. Numerous studies carried out in different parts of the world have reported similar findings during classroom occupancy [31,34,41,43,106]. The largest particles in this study were found to be the highest contributor to indoor aerosol mass concentrations. Indoor PM_10_ concentrations of up to 5 times (230 µg m^−3^) higher than outdoor air were registered, while similar indoor and outdoor concentrations were observed for PM_2.5_ (~41 µg m^−3^) (Table S3). The highest indoor/outdoor (I/O) ratios for PM_10_ (close to 3) and PM_2.5_ (close to 2) were observed at midday when students started workout activities inside the classroom. Similar patterns were observed in other studies aimed at studying air quality in schools. Rivas et al. [40] reported that indoor PM_2.5_ concentrations were almost twice the outdoor values in Barcelona. Chithra and Nagendra [59] found an I/O ratio of 2.5 for PM_10_ and 1.4 for PM_2.5_ in Chennai (India). Stranger et al. [34] reported PM_2.5_ I/O ratios in primary schools in Belgium higher than 1 (range 1.3–2.3). Faria et al. [30] studied the PM_10_ and PM_2.5_ in five schools in Lisbon, reporting I/O ratios of 1.8 and 2.1, respectively, during occupancy, which is in line with the findings also obtained in Lisbon by Martins et al. [60]. A study in Hangzhou (China) also reported strongly enhanced PM_10_ levels in a primary school during daytime student activities, up 500% compared with the non-occupancy period [32]. In this case, this was the result of particle resuspension due to students’ movements and the use of chalk in the classroom.

Unlike other studies [85,107,108,109], daily exceedances of WHO [4] and U.S. EPA [110] PM_10_ (45 and 150 µg m^−3^, respectively) and PM_2.5_ (15 and 35 µg m^−3^, respectively) outdoor standards were not recorded during the measurement period. Elevated indoor PM_x_ concentrations in primary school classrooms raise concerns about possible adverse health effects on children because exposure to high levels of PM_x_ in classrooms is associated with respiratory [14,17,18] and skin [19] problems.

PM_1_ concentrations were less influenced by the anthropogenic activities inside the classroom, being only the mean PM_1_ I/O ratio of 0.8 (Table S3). PM_1_ contributed more to PM_10_ at night (89%) and morning (70%) when the classroom was empty (Figure S1 and Table S3), showing a similar trend both for indoor and outdoor air. This indicated that the particles < 1 µm showed almost no resuspension, as some studies have found (e.g., [111,112]). In the absence of noticeable indoor sources of ultrafine particles in the classroom, particles < 1 µm are of outdoor origin, in particular from traffic emissions, as already discussed in the previous section. This has also been observed in other schools with comparable environments [59]. A similar situation occurred for PNC. Its concentration did not show variations associated with classroom occupancy; on average, the I/O ratio was 0.3. This confirms the absence of indoor sources for PNC. In the case of eBC, although it is a traffic-related pollutant, the high I/O ratio, on average, was the result of its special behavior, specifically its low diffusivity capacity [91].

The most common and easy method for air renewal in indoor environments is natural ventilation by the temporary opening of windows. When windows are opened for a few hours, the I/O ratios of PNC and eBC increase, as other studies have also shown [99]. In this regard, the ventilation mode determines the pollutant’s infiltration and, thus, its influence on the I/O ratios [99,113,114]. For example, in this study, eBC concentration, when windows were opened on 13 December from 10:30 to 11:30 UTC, varied from 2.4 to 5.7 µg m^−3^, and PNC, on 15 December from 09:10 to 09:30 UTC, ranged from 3500 to 6560 particles cm^−3^. Thus, the highest I/O ratios for these pollutants were found in the classroom ventilated naturally with open windows. In this study, indoor PM_x_ levels in the classroom did not seem to be influenced by opening windows.

Although indoor/outdoor pollutant measurements for the school are not available in the warm period, some studies have analyzed window-opening periods’ influence on indoor air quality during cold and warm months. The differences found are related to the window opening time, the pollutants (indoor or outdoor origin) measured, and their concentrations. For example, Stabile et al. [115] identified that naturally ventilated Italian schools affect indoor pollutants differently, reducing concentrations of indoor-origin pollutants, but increasing those concentrations of outdoor-origin pollutants (especially traffic-related pollutants) measured indoor. A similar finding was observed by Fuoco et al. [116] in three public primary schools in Cassino (Central Italy), longer airing periods resulted in a higher outdoor pollutant infiltrate, with I/O ratios being higher in warm months. Matthaios et al. [10] observed a positive association between indoor exposure related to outdoor pollutants and the number of open windows in classrooms in urban schools in the Northeast United States. Similarly, Baloch et al. [117] found that the level of pollutants from outside measured inside classrooms was worse during the warm season as a consequence of inadequate ventilation. In Madrid, emissions from traffic decrease in warm months [66]. In addition, an increase in temperature and solar irradiance leads to intense convective processes and regional air flows, and well-formed recirculation processes promote greater pollutant dispersion [118]. Thus, opening windows may similarly affect indoor air quality over the cold period, increasing traffic-related pollutants indoors, but with lower concentrations. Thus, inadequate ventilation rates in classrooms could worsen indoor air quality.

In Figure 6, the increase in indoor PM_x_ levels observed on 15 December after the windows were closed possibly corresponds to the classroom occupancy, although this activity was not recorded.

Occupancy data correspond to two different periods, one of them with ventilated atmospheric conditions (11–15 December 2017) and the other with poor atmospheric dispersion (non-ventilated) (18–20 December 2017) (Table S3). I/O ratios for the pollutants measured were higher during the ventilated instability period, especially for PM_10_ and PM_2.5_. This period included a greater number of days than the non-ventilated stability period and the opening of windows was less frequent, probably resulting in higher concentrations of indoor pollutants (Figure S1).

During the post-occupancy period, the pollutant levels in the classroom gradually returned to the previous values.

## 3. Conclusions

The present study characterizes, for the first time, in Madrid the indoor air quality (IAQ) in a primary school located in a traffic-influenced urban area. To this end, simultaneous indoor/outdoor (I/O) measurements of PM-related pollutants (PM_10_, PM_2.5_, PM_1_, ultrafine particle number concentration (PNC), and equivalent black carbon (eBC)) were obtained at different sampling points inside (classrooms) and outside of the school under different meteorological and air pollution conditions.

A good mixing of the ambient (outdoor) air was found over a period of the study. Outdoor PNC and eBC concentrations had similar variation patterns at the measurement points, reaching the highest levels during the traffic rush hours. This indicated that both pollutants are related to traffic as the main source. Outdoor pollutant levels were typically several times higher than indoors. However, this situation was disturbed under specific meteorological conditions and during some students’ activities in the classrooms.

In the absence of activity in the classroom, indoor pollutants behaved similarly to outdoor ones, especially PNC and eBC, suggesting that the indoor levels were strongly influenced by traffic emissions of the school surroundings. These pollutants are reported by the World Health Organization (WHO) as a universal carrier of a wide variety of toxic chemicals to humans [4] and associated with adverse health effects. On the other hand, eBC I/O ratio was higher than PM_x_ and PNC during the non-occupancy periods, finding values higher than 1. This could be the result of the particle diffusion factor and the penetration efficiency, which varied with the particle size. I/O ratios strongly depended on meteorological factors, showing statistically significant differences between meteorological periods (with *p*-values between <2 × 10^−16^ and 2.14 × 10^−6^). Consequently, the meteorology (specifically the wind speed) influenced the time delay variability of indoor vs. outdoor pollutants, finding an inversely proportional to wind speed.

Resuspension of indoor accumulated dust was the main source of pollutants in classrooms during the occupied periods, resulting in the highest I/O ratios for particle sizes greater than 1 µm during the study period. However, exceedances of 24 h WHO and US-EPA standards PM_10_ and PM_2.5_ for indoor air quality did not occur. PM_1_ was less significantly affected by occupancy. This suggested a different origin, possibly from outdoor sources. In addition, opening windows leads to an increase in PNC and eBC indoor levels. Identifying the high concentrations of traffic-related pollutants in classrooms has been associated with respiratory problems (asthma, allergies, or rhinoconjunctivitis [12,17,18]), dermal symptoms [19], children’s well-being [20], loss of students’ attention and academic achievement [24], or neurobehavioral disorders [26].

Given that children are one of the most vulnerable population groups in our society, assessing their exposure to indoor air pollutants in schools is of special interest for proposing ad hoc measures for improving indoor air quality at schools and reducing the pollutant’s exposure in these environments and the children’s health problems that derive from this. Thus, from the observations and conclusions obtained in this study, the following recommendations are proposed for schools situated in an area with high-traffic density: (i) reduction in traffic around school environments, (ii) implementation of a green transportation program, (iii) classroom ventilation during periods of high outdoor air quality, and (iv) maintain proper cleaning of classrooms and the school environment. This information could help to establish new legislation about pollutant parameters and standards as well as the typology of the indoor spaces and building recommendations. The lack of summer information on indoor/outdoor air quality and meteorology in the study area will be addressed in a future investigation with a greater number of schools influenced by different factors and types of sources to understand how the I/O sources affect indoor air quality, the pollutant’s atmospheric transport, and infiltration mechanisms and their control.

## Figures and Tables

**Table 1:** Sampling points and measuring instruments, parameters, and features. The measurement period is indicated for each instrument. “IN” refers to the indoor measurements and “OUT” to the outdoor ones.

Sampling Point	Instrument	Parameters	Measurement Size Range (µm)	Measurement Period
Classroom A	2 MicroAeth^®^ model AE51 (IN + OUT)	eBC	Total	27 November–21 December 2017
	CPC, TSI model 3772 (IN)	PNC	>0.01	22 November–21 December 2017
	CPC, TSI models 3775 and 3772-CEN (OUT)		>0.004	
	2 GRIMM Mini-Las 11-R (IN + OUT)	PM_10_, PM_2.5_, and PM_1_ obtained from counts	Mass concentration of particles <10, <2.5, and <1 µm, respectively	5–21 December 2017
Classroom B	2 MicroAeth^®^ model AE51 (IN + OUT)	eBC	Total	15–20 December 2017
External check-point	MicroAeth^®^ model AE51 (OUT)	eBC	Total	5–20 December 2017
	CPC, TSI model 3775 (OUT)	PNC	>0.004	15–16 December 2017
	Air quality measurements (OUT)	Trace gases (SO_2_, NO, NO_2_ and O_3_) and PM_10_	-	22 November–21 December 2017
CIEMAT site	Permanent meteorological tower	AP, T at two heights, RH, WS, WD	-	22 November–21 December 2017

**Table 2:** Indoor/outdoor (I/O) ratios for all pollutants (PNC, PM_10_, PM_2.5_, PM_1_, and eBC) measured in the two classrooms (A and B) during the non-occupancy (weekends and vacation) for atmospheric stability (from 6 (00:00 UTC) to 9 December 2017 (11:00 UTC)) and instability (from 16 (00:00 UTC) to 17 December 2017 (21:00 UTC)) conditions. Here, different periods are considered: at night (00:00–04:00 UTC), morning (05:00–09:00 UTC), midday (11:00–15:00 UTC), and evening (17:00–21:00 UTC). Wind speed (WS) values are included as a proxy of ventilation conditions during each period. The geometric mean was used to calculate the mean I/O ratio from 10 min data of the pollutants measured in this study.

Parameters		Mean	Night	Morning	Midday	Evening
**Atmospheric Stability Situation**						
WS (m s^−1^)		1.3	1.2	1.1	1.5	1.6
Classroom A						
I/O ratios	PNC					
	PM_10_	0.4	0.5	0.4	0.4	0.3
	PM_2.5_	0.5	0.6	0.5	0.5	0.4
	PM_1_	0.5	0.7	0.6	0.5	0.4
	eBC					
**Atmospheric Instability Situation**						
WS (m s^−1^)		3.4	4.1	1.7	3.2	4.2
Classroom A						
I/O ratios	PNC	0.2	0.2	0.1	0.2	0.2
	PM_10_	0.6	0.6	0.4	0.9	0.5
	PM_2.5_	0.7	0.6	0.4	1.1	0.5
	PM_1_	0.7	0.8	0.5	1.2	0.6
	eBC	1.2	0.7	0.7	2.4	1.0
Classroom B						
I/O ratios	eBC	0.8	0.7	0.7	1.3	0.6

## Data Availability

The data that support the findings of this study are available from Área de Gobierno de Medio Ambiente y Movilidad of the Ayuntamiento de Madrid, but restrictions apply to the availability of these data, which were used under license for the current study, and so are not publicly available. Data are, however, available from the authors upon reasonable request and with the permission of Área de Gobierno de Medio Ambiente y Movilidad of the Ayuntamiento de Madrid.

## Supplementary Materials

The following supporting information can be downloaded at: https://www.mdpi.com/article/10.3390/ijerph21101263/s1, Figure S1: Simultaneous time series of indoor/outdoor (I/O) pollutants (PNC, PM_10_, PM_2.5_, PM1, and eBC) measured in classrooms A and B. In the graph, the prefix “Out.” refers to outdoor measurements represented with solid areas, whereas “In.” indicates indoor measurements represented with lines. The lower panel corresponds to eBC measurements in classroom B, whereas the first five panels above are measurements taken in classroom A. Periods of different activities (occupancy, open windows, or both) are indicated, as well as weekends; Table S1: Descriptive statistics (mean (sd) and percentiles (50th and 98th)) of meteorological parameters (T = temperature, RH = relative humidity, WS = wind speed, and P = accumulated precipitation) and air quality pollutants (PM_10_, SO_2_, NO, NO_2_, and O_3_) registered during the different atmospheric conditions that occurred in the measurement period. “Stability” refers to atmospheric stability situations, “Intermediate” to intermediate atmospheric situations, and “Instability” to atmospheric instability situations; Table S2: Descriptive statistics (maximum, minimum, mean (sd), and median) for all pollutants (PNC, PM_10_, PM_2.5_, PM_1_, and eBC) measured in the two classrooms during the non-occupancy (weekends and vacation) for each meteorological situation at night (00:00–04:00 UTC), morning (05:00–09:00 UTC), midday (11:00–15:00 UTC), and evening (17:00–21:00 UTC) periods; Table S3: Descriptive statistics (maximum, minimum, mean (sd), and median) and hourly indoor/outdoor (I/O) ratios for all pollutants (PNC, PM_10_, PM_2.5_, PM_1_ and eBC) measured in classroom A during the occupancy period at night (Nig., 00:00–04:00 UTC), morning (Mor., 05:00–09:00 UTC), midday (Mid., 11:00–15:00 UTC), and evening (Eve., 17:00–21:00 UTC) periods. Hourly indoor/outdoor (I/O) ratios for the pollutants considered were included. The geometric mean was used to calculate the mean I/O ratio of the pollutants measured in this study; Table S4: ANOVA test result for the indoor/outdoor (I/O) ratios during the atmospheric stability and instability for all pollutants (PNC, PM_10_, PM_2.5_, PM_1_, and eBC) measured in the two classrooms (A and B) during the non-occupancy (weekends and vacation).
