# Supplementary material for: Indoor Air Quality at an Urban Primary School in Madrid (Spain): Influence of Surrounding Environment and Occupancy
Source: Int J Environ Res Public Health. 2024 Sep 24;21(10):1263. doi: 10.3390/ijerph21101263 (PMC11506914; doi:10.3390/ijerph21101263)
Supplement: Supplementary file 1 [file ijerph-21-01263-s001.zip › ijerph-3139766-supplementary.pdf]

Supplementary Materials

Indoor air quality at an urban primary school in Madrid (Spain): Influence of surrounding environment and occupancy

E. Alonso-Blanco[1], F.J. Gómez-Moreno[1], E. Díaz-Ramiro[1], M. Barreiro[1], J. Fernández[1], I. Figuero[1], A. Rubio-Juan[2], J.M. Santamaría[3] and B. Artíñano[1]

[1] Center for Energy, Environmental and Technological Research (CIEMAT), Avenida Complutense 40, 28040 Madrid, Spain.

[2] Centro Regional de Selección y Reproducción Animal (CERSYRA). Consejería de Agricultura y Medio Ambiente de Castilla-La Mancha. Avenida del Vino, 2. 13300-Valdepeñas. Ciudad Real, Spain.

[3] Integrated Laboratory for Environmental Quality (LICA), Department of Chemistry, University of Navarra, Irunlarrea No 1, 31008 Pamplona, Spain.

Correspondence to: E. Alonso-Blanco (elisabeth.alonso@ciemat.es)

Figures

Figure S1. Simultaneous time series of indoor/outdoor (I/O) pollutants (PNC, PM_{10}, $PM_{2.5}$, PM_1 and eBC) measured in classrooms A and B. In the graph the prefix “Out.” refers to outdoor measurements represented with solid areas, whereas “In.” indicates indoor measurements represented with lines. The lower panel corresponds to eBC measurements in classroom B, whereas the first five panels above are measurements taken in classroom A. Periods of different activities (occupancy, open windows or both) are indicated, as well as weekends.

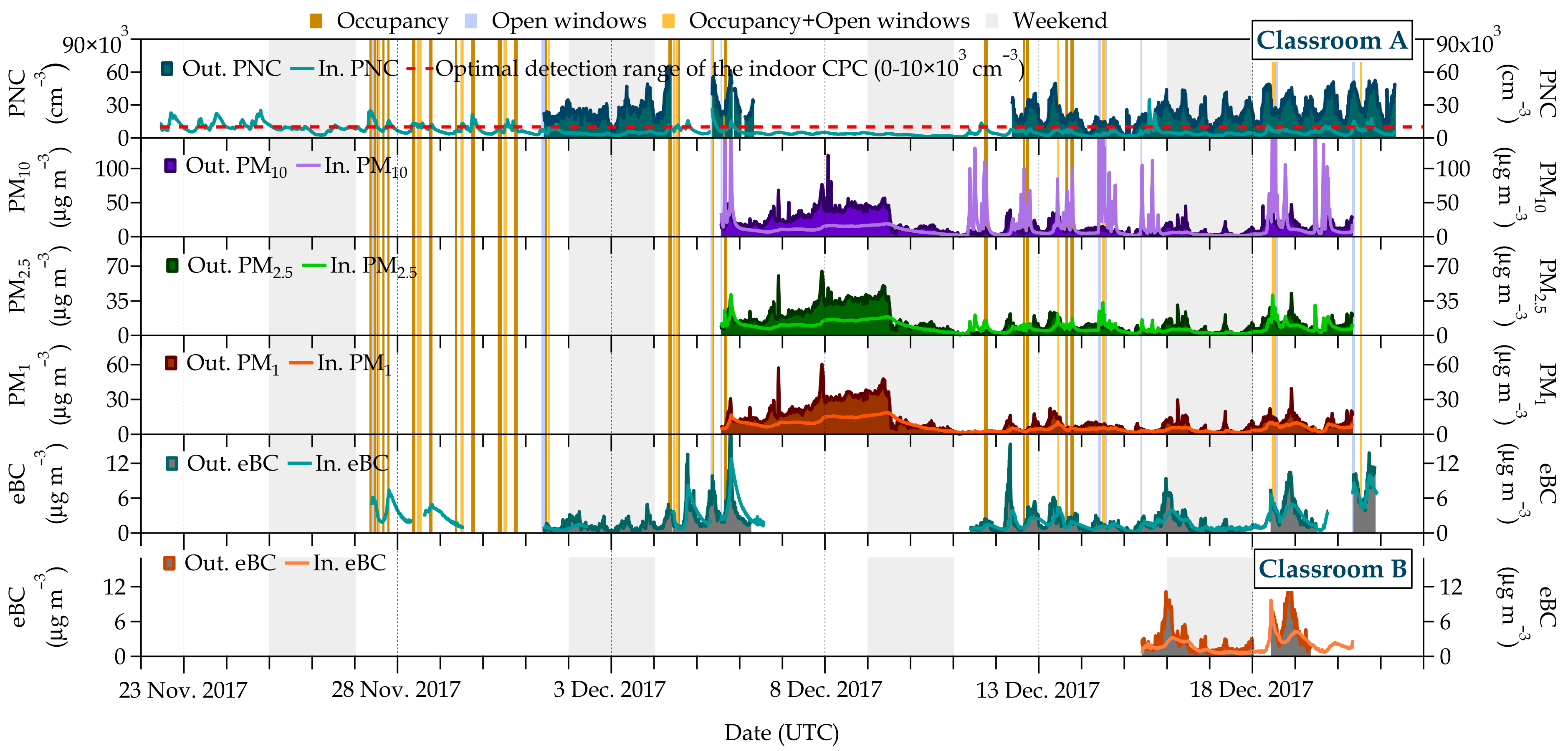

Occupancy
Open windows
Occupancy+Open windows
Weekend
Classroom A
Out. PNC
In. PNC
Optimal detection range of the indoor CPC (0-10×10^3 cm^-3)
PNC (cm^-3)
Out. PM10
In. PM10
PM10 (µg m^-3)
Out. PM2.5
In. PM2.5
PM2.5 (µg m^-3)
Out. PM1
In. PM1
PM1 (µg m^-3)
Out. eBC
In. eBC
eBC (µg m^-3)
Classroom B
23 Nov. 2017
28 Nov. 2017
3 Dec. 2017
8 Dec. 2017
13 Dec. 2017
18 Dec. 2017
Date (UTC)

Tables

Table S1. Descriptive statistics (mean(sd) and percentiles (50th and 98th)) of meteorological parameters (T=temperature, RH=relative humidity, WS=wind speed and P=accumulated precipitation) and air quality pollutants (PM_{10}, SO_2, NO, NO_2 and O_3) registered during the different atmospheric conditions that occurred in the measurement period. "Stability" refers to atmospheric stability situations, "Intermediate" to intermediate atmospheric situations and "Instability" to atmospheric instability situations.

Atmospheric Situations	Date and time (UTC)	T (°C)	RH (%)	WS (m s^{-1})	P (mm)	PM_{10} (µg m^{-3})			SO_2 (µg m^{-3})			NO (µg m^{-3})			NO_2 (µg m^{-3})			O_3 (µg m^{-3})		
						Mean (sd)	P50	P98	Mean (sd)	P50	P98	Mean (sd)	P50	P98	Mean (sd)	P50	P98	Mean (sd)	P50	P98
Stability	22 (00:00)-25.11.2017 15:00	11.1(3.3)	61.8(16.3)	1.9(1.1)	0.1	51(17)*	48	101	10(6)	8	26	131(101)	108	414	92(39)*	85	205	9(13)	3	46
Intermediate	25 (17:00)-26.11.2017 16:00	7.5(3.6)	61.7(15.0)	3.9(1.8)	0.0	7(5)	7	21	3(1)	3	6	5(4)	4	17	21(21)	19	21	44(11)	47	59
	26 (17:00)-29.11.2017 09:00	4.7(2.8)	72.2(18.2)	1.7(1.2)	2.2	33(16)	28	69	8(5)	7	24	97(69)	78	257	74(22)	72	125	5(5)	3	25
	29.11.2017 10:00-03.12.2017 18:00	4.3(2.9)	53.0(12.3)	3.9(2.0)	0.0	11(5)	10	25	4(2)	4	12	16(25)	8	107	43(24)	34	95	37(21)	44	64
	All Period	4.8(3.1)	60.5(17.2)	3.2(2.0)	2.2	18(15)	12	67	6(4)	4	16	42(59)	11	213	50(28)	50	108	27(23)	26	62
Stability	03 (18:00)-09.12.2017 11:00	4.5(3.8)	65.1(12.8)	1.6(0.9)	0.0	32(16)*	29	70	10±5	9	22	99(82)	72	344	78(31)*	71	165	7(10)	3	44
Instability	09 (12:00)-17.12.2017 21:00	6.7(3.3)	73.8(17.2)	4.4(2.6)	10.6*	13(10)	10	46	4±3	3	13	28(55)	6	215	41(29)	35	113	31(21)	29	70
Stability	17 (22:00)-22.12.2017 00:00	6.3(3.8)	63.5(15.6)	1.8(1.3)	0.6	30(17)*	24	79	9±5	8	20	119(103)	81	423	86(36)*	80	179	9(13)	4	53

* The asterisk indicates the most the significant results.

Table S2. Descriptive statistics (maximum, minimum, mean(sd) and median) for all pollutants (PNC, PM_{10}, $PM_{2.5}$, PM_1 and eBC) measured in the two classrooms during the non-occupancy (weekends and vacation) for each meteorological situation at night (00:00-04:00 UTC), morning (05:00-09:00 UTC), midday (11:00-15:00 UTC) and evening (17:00-21:00 UTC) periods.

	Indoor								Outdoor							
	Max.	Min.	Mean(sd)	Median	Night	Morning	Midday	Evening	Max.	Min.	Mean(sd)	Median	Night	Morning	Midday	Evening
Atmospheric Stability Situation (6 (00:00 UTC)-9.12.2017 11:00 UTC)																
Classroom A																
PNC (cm^{-3})	6109	2869	4097(628)	3928	3996	3230	4131	4072								
PM_{10} ($\mu g\ m^{-3}$)	19	7	13(3)	11	13	12	12	11	119	14	35(13)	37	29	29	32	40
$PM_{2.5}$ ($\mu g\ m^{-3}$)	19	7	13(3)	11	12	12	12	11	65	11	29(11)	31	23	25	26	31
% of PM_{10}			99%		99%	99%	98%	99%			81%		79%	85%	81%	79%
PM_1 ($\mu g\ m^{-3}$)	19	6	12(3)*	10	12	12	11	11	60	10	26(10)*	28	20	22	24	27
% of PM_{10}			95%		95%	96%	95%	95%			73%		71%	76%	73%	70%
eBC ($\mu g\ m^{-3}$)																
Atmospheric Instability Situation (16 (00:00 UTC)-17.12.2017 21:00 UTC)																
Classroom A																
PNC (cm^{-3})	5562	2156	3694(831)	3639	3609	3275	4098	3457	43711	6143	20790(8446)	19185	18093	23525	17702	22985
PM_{10} ($\mu g\ m^{-3}$)	7	1	3(2)	3	4	4	4	2	45	1	7(6)	4	9	10	5	5
$PM_{2.5}$ ($\mu g\ m^{-3}$)	7	1	3(2)	3	4	4	4	2	31	1	6(5)	4	7	9	4	4
% of PM_{10}			98%		98%	99%	97%	99%			84%		83%	88%	82%	87%
PM_1 ($\mu g\ m^{-3}$)	6	1	3(2)*	3	3	4	3	2	30	1	5(4)*	3	6	8	3	3
% of PM_{10}			90%		89%	91%	88%	91%			72%		69%	78%	68%	72%
eBC ($\mu g\ m^{-3}$)	1.4	0.4	1.4(1.0)	0.8	2.1	1.6	1.3	0.7	1.5	0.2	1.5(1.6)	0.6	5.6	1.0	0.9	0.6
Classroom B																
eBC ($\mu g\ m^{-3}$)	1.5	0.4	1.5(1.0)	1.0	2.0	1.8	1.6	0.8	2.1	0.4	2.1(2.1)	1.2	7.4	1.5	1.3	1.2

* The asterisk indicates the most the significant results.

Table S3. Descriptive statistics (maximum, minimum, mean(sd) and median) and hourly indoor/outdoor (I/O) ratios for all pollutants (PNC, PM_{10}, $PM_{2.5}$, PM_1 and eBC) measured in classroom A during the occupancy period at night (Nig., 00:00-04:00 UTC), morning (Mor., 05:00-09:00 UTC), midday (Mid., 11:00-15:00 UTC) and evening (Eve., 17:00-21:00 UTC) periods. Hourly indoor/outdoor (I/O) ratios for the pollutants considered have been included. The geometric mean has been used to calculate the mean I/O ratio of the pollutants measured in this study.

	Indoor								Outdoor								Indoor/Outdoor (I/O) ratios							
	Max.	Min.	Mean(sd)	Median	Nig.	Mor.	Mid.	Eve.	Max.	Min.	Mean(sd)	Median	Nig.	Mor.	Mid.	Eve.	Max.	Min.	Mean	Median	Nig.	Mor.	Mid.	Eve.
PNC (cm^{-3})	34940	931	5387(3250)	4918	2822	3992	6319	7948	52436	1176	23174(12137)	20786	12874	27067	19534	30190	3.2	0.1	0.3*	0.2	0.3	0.2	0.3	0.3
PM_{10} ($\mu g\ m^{-3}$)	230	1	19(28)*	8	5	6	38	31	47*	1	11(8)	9	8	14	11	12	19.5	0.1	1.1	0.9	0.7	0.4	2.4*	2.3
$PM_{2.5}$ ($\mu g\ m^{-3}$)<br>% of PM_{10}	41	1	7(5)<br>38%	6	5<br>98%	5<br>81%	11<br>29%	10<br>30%	43*	1	8(6)<br>74%	7	7<br>85%	11<br>77%	7<br>64%	8<br>71%	8.1	0.2	0.9	0.9	0.8	0.5	1.6*	1.2
PM_1 ($\mu g\ m^{-3}$)<br>% of PM_{10}	11	1	4(2)*<br>23%	4	4*<br>89%	4*<br>70%	5<br>12%	5<br>16%	39	0	7(5)<br>61%	6	6<br>72%	9<br>65%	5<br>50%	7<br>57%	5.3	0.2	0.8	0.8*	0.9	0.5	1.0	0.8
eBC ($\mu g\ m^{-3}$)	9.9	0.1	2.2(1.9)	1.5	1.6	1.5	2.2	3.0	15.3	0.1	2.7(2.7)	1.6	1.0	3.2	2.2	3.9	4.9	0.1	1.0	1.0	2.0	0.5	1.1	0.8

* The asterisk indicates the most the significant results.

Table S4. ANOVA test result for the indoor/outdoor (I/O) ratios during the atmospheric stability and instability for all pollutants (PNC, PM_{10}, $PM_{2.5}$, PM_1 and eBC) measured in the two classrooms (A and B) during the non-occupancy (weekends and vacation).

			Degrees of freedom (residuals)	Sum of Freedom (residuals)	Mean Squares (residuals)	F-value	p-value
Classroom	A	PNC	1 (1828)	32.0 (368.6)	31.97 (0.20)	158.6	$<2\times10^{-16}$
		PM_{10}	1 (2119)	157.7 (1586.2)	157.69 (0.75)	210.7	$<2\times10^{-16}$
		$PM_{2.5}$	1 (2119)	93.8 (709.3)	93.81 (0.33)	280.2	$<2\times10^{-16}$
		PM_1	1 (2119)	76.8 (478.2)	76.80 (0.23)	340.3	$<2\times10^{-16}$
		eBC	1 (1526)	8.4 (569.3)	8.447 (0.373)	22.64	2.14×10^{-6}
	B	eBC	1 (506)	10.41 (180.86)	10.413 (0.357)	29.13	1.04×10^{-7}
